# A Step towards Concrete with Partial Substitution of Waste Glass (WG) in Concrete: A Review

**DOI:** 10.3390/ma15072525

**Published:** 2022-03-30

**Authors:** Jawad Ahmad, Zhiguang Zhou, Kseniia Iurevna Usanova, Nikolai Ivanovich Vatin, Mohammed A. El-Shorbagy

**Affiliations:** 1 Department of Disaster Mitigation of Structures, Tongji University, Shanghai 200092, China; jawadcivil13@scetwah.edu.pk; 2 Peter the Great Saint Petersburg Polytechnic University, 195251 Saint Petersburg, Russia; plml@mail.ru (K.I.U.); vatin@mail.ru (N.I.V.); 3 Department of Mathematics, College of Science and Humanities in Al-Kharj, Prince Sattam bin Abdulaziz University, Al-Kharj 11942, Saudi Arabia; ma.hassan@psau.edu.sa

**Keywords:** sustainable concrete, waste glass, mechanical performance, durability, microstructure analysis

## Abstract

The annual worldwide production rate of waste glass is a million tons; the waste glass is non-biodegradable, resulting in environmental pollution. However, the chemical composition of waste glass (WG) is promoted to be used as a partial substitution of binding or filler (aggregate) material in concrete production. Although significant research has been conducted in this area, the results of these studies are scattered, and it is difficult to judge the suitability of waste glass in concrete. This review looks at the effects of waste glass on concrete’s fresh, mechanical, and durability properties. It concludes that waste glass decreased the flowability of concrete. Furthermore, waste glass can be used as pozzolanic material, creating secondary cementitious compound (CSH) gel. CSH gel increased the cement paste’s binding properties, leading to increased mechanical performance. Moreover, this study reveals that the optimum dose of waste glass is important to minimize the possibility of an alkali–silica reactions. Based on this review, most researchers conclude that 20% substitution of waste glass as binding material is the optimum dose. The wide range of discussion provides the necessary guideline for the best research practice in the future.

## 1. Introduction

**Figure 1:**
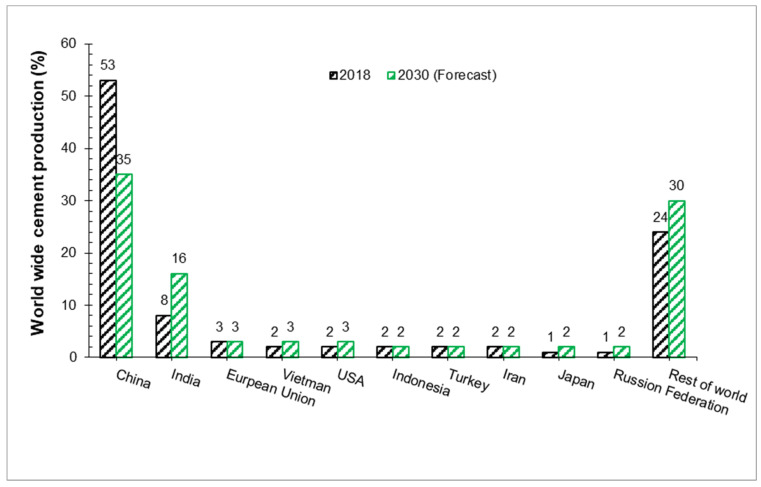
Worldwide cement production 2018 and forecast 2030. Data source: World Cement Association [1].

Figure 1 shows the worldwide cement production rate according to the world cement association conference [1]. It can be observed that cement production has been increasing continuously from 1990 to 2030. Its manufacture is rapidly increasing, especially in developing countries, such as China, Russia, and Japan, due to the need for cement for modern housing and infrastructure [2]. The cement industry has now built the capacity to generate 59.5 million tons due to the expectation of the potential demand for developing infrastructure. In the short time of 10 years, cement cost has increased to almost 150% [3]. Cement is most costly material in plain cement concrete as compared to the other ingredients of concrete. Therefore, it is important to search other supplementary materials instead of cement.

Sustainable construction usage means responsible management for producing a beneficial environment while overseeing ecology and the development of resources [4]. Cement concrete is a major construction material globally, and it is inexpensive while possessing better performance than other materials. However, it has effects on the ecosystem [5,6,7,8,9,10]. Producing cement, which is a major ingredient of concrete, is a major source of discharges of greenhouse gases CO_2_. Each year, the world presently manufactures about 3.6 billion metric tons of the material [11]. In 2030, the volume is expected to increase to more than 5 billion metric tons [12,13]. Even though the status is different from each country, about half of the world’s ordinary Portland cement (OPC) produces 11 billion metric tons of concrete yearly; the rest is used in screeds, mortars, soil stabilization, coatings, and other applications [14]. To reduce such an amount of CO_2_, it is important to incorporate waste materials in concrete instead of cement.

Presently, cement manufacturing causes more than 5% of global CO_2_ emissions [15]. CO_2_ emissions can be decreased by substituting OPC with cementation materials [16,17,18]. Byproducts of the industry can be used in multi-component binder materials for a wide range of applications [19]. Several industrial wastes are utilized effectively in binding material, including waste marble, waste foundry sand, fly ash, granulated blast furnace slag, rubber, etc. [13,20,21,22,23,24,25]. Various research studies proposed to make concrete by using supplementary material to decrease cost and shortage of standard materials [26]. The practice of using waste in concrete becomes economical, and reusing waste is the greatest environmental choice for taking on the care of garbage dumping [27].

In contrast, the amount of waste generated by the industry has increased worldwide due to increased demand and product utilization. Only a small amount has been utilized, and the remainder has been trashed indiscriminately, causing environmental problems. The tremendous increase in the amount of garbage that must be discarded, the scarcity of discarding locations, and the quick rise in transportation and discarding expenses all have an adverse effect on the environment, placing a halt to sustainable growth. The hindrance of waste removal is becoming intense [28]. The total global glass waste preparation estimate was 130 Mt beginning in 2005. China, the United States, and the European Union produce around 32 Mt, 20 Mt, and 33 Mt, respectively [29]. Glass disposal as a landfill that has environmental difficulties and can be costly because it is inherently non-biodegradable [30,31,32]. It is abundant, has a minimal economic value, and frequently creates landfills [33]. Investigations also considered utilizing glass waste as aggregates in the manufacture of concrete [34,35]. Glass pozzolanic properties initially became apparent at particle sizes less than 100 microns m and less than 300 microns m, respectively [36]. According to past studies, glass powder increases the mechanical performance of concrete [37,38,39]. Grinding might increase the pozzolanic reactivity of secondary cementitious materials (SCMs) [40,41]. Because of their low hydration temperatures, pozzolanic cement mixes are resistant to thermal cracking [42,43,44]. A good pozzolanic attempt to avoid alkali–silica interaction uses lime to significantly reduce efflorescence [25]. It also reported that cathode ray tube waste glass as fine aggregates could be utilized in self-compacting concrete [45].

Currently, the uses of waste glass in concrete production are being extended, involving precast concrete blocks, road paving blocks, marine structures, precast concrete slabs, and blocks [46]. According to the authors’ best knowledge, most researchers focus on fly ash, silica fume, etc., as a pozzolanic material, while a compressive review on waste glass is limited [47,48]. However, less information is available regarding durability and particular ASR, which is one of the big challenges for used waste glass in concrete. This review focuses on the mechanical durability ASR of concrete with waste glass, which will also provide ideas for a new researcher in choosing and applying waste glass. The current review studies the different methods of using waste glass in concrete and investigates the effect of the substitution ratio and particle size on the mechanical durability ASR of concrete. A successful review provides the necessary guideline for readers and the best research practices in the future.

## 2. Role of Waste Glass

**Figure 2:**
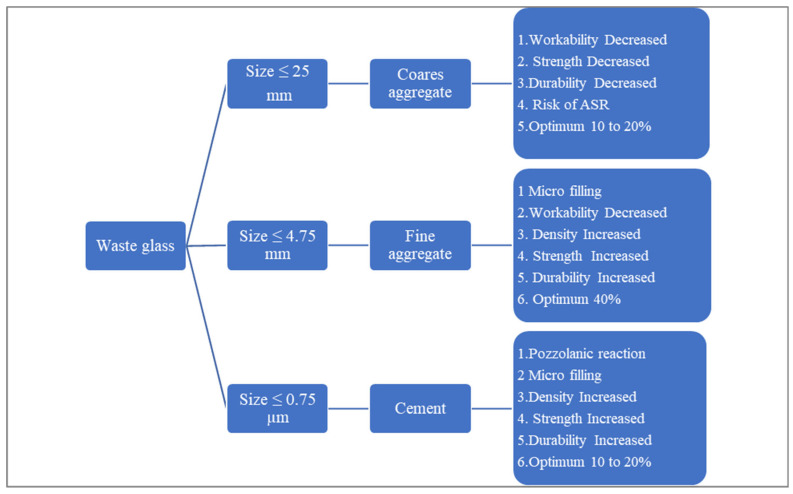
Role of waste glass in concrete.

Waste glass can be used as a cement replacement or as filler material (aggregate) in cement concrete production. However, the results depend on the particle size of waste glass, substitution rate, and chemical composition, which must be considered during the mix design. Figure 2 shows the role of waste glass in concrete.

## 3. Chemical Composition of Waste Glass

**Figure 3:**
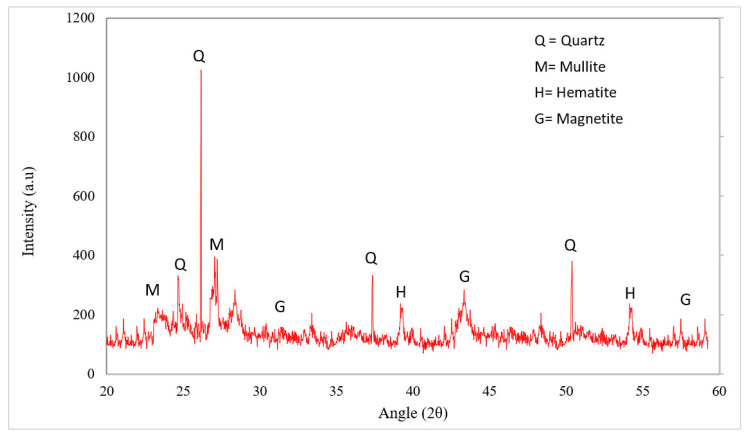
XRD analysis of waste glass [56].

Mechanical and durability performance of concrete with waste glass also depends on the percentage of different chemicals present in waste glass. A study reported that the pozzolanic activity of waste glass mainly depends on chemical composition and particle size [49]. Table 1 shows the chemical composition of waste glass as per past studies. It can be observed that the main chemical composition of waste glass lies in the range of SiO_2_ (70 to 75%), CaO (8 to 10%), Al_2_O_3_ (1 to 3%), MgO (1.5 to 3%), NaO_2_ (0 to 15%), and Fe_2_O_3_ (0.5 to 1%) [50,51,52]. The amorphous nature of SIO_2_ present in waste glass plays a vital role in the performance of concrete, starting from hydration up to the final development of strength [49]. According to ASTM [53], the accumulation of a chemical (SiO_2_, CaO, Al_2_O_3_, MgO, NaO_2,_ and Fe_2_O_3_) up to more than 70% can be used as pozzolanic material waste glass containing more than 70%. Figure 3 shows the XRD results of waste glass. A major peak of quartz (SiO_2_) was observed at 27-degree angles. Some minor peaks were also observed at different angles, which shows the amorphous nature of waste glass. A study concluded that the peak heat flow during the hydration process and Ca(OH)_2_ decreased with the substitution of waste glass as a binding material, which undoubtfully improved mechanical performance [54]. However, the pozzolanic activity of waste glass is mainly dependent on particle size. A study was carried out on different particle sizes of waste glass (300 to 38 mm) and the pozzolanic activity of waste glass on the particle size below 150 mm was observed. Furthermore, a study concluded that the pozzolanic activity of waste glass increased with the decreasing particle size of waste glass. The maximum pozzolanic activity was observed at 38 mm particle size of waste glass. Moreover, it also indicated that higher doses of waste glass can cause alkali silica reactions (ASR) due to a deficiency of calcium hydrate [49]. It has been also reported that higher doses of waste glass decreased the mechanical performance of concrete due to dilution effects, which caused the alkali silica reaction [55]. Therefore, it important to choose the optimum dose of waste glass.

## 4. Fresh Properties

### 4.1. Slump Cone

**Figure 4:**
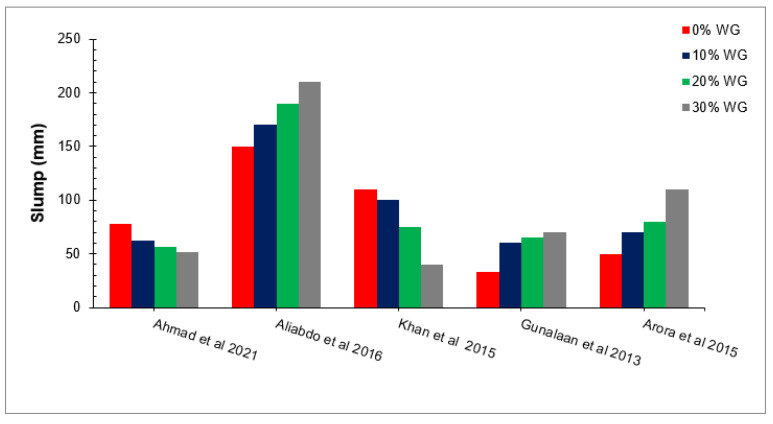
Slump with different percentages of waste glass [49,60,61,62,63].

Concrete workability is a broad and subjective term that describes how easily freshly concrete can be mixed, placed, consolidated, and finished while maintaining homogeneity. A summary of slump flow with different percentages of waste glass (0 to 30% in increment of 10%) according to the paste studies is shown in Figure 4 [49,60,61,62,63]. Workability is one of the most important properties of fresh concrete, which influences concrete’s mechanical and durability performance [64]. The mechanical and durability of concrete depends on the flowability of fresh concrete. The lack of flowability increases compaction efforts, resulting in pores in hardened concrete. The increase in voids decreases the concrete density of concrete, resulting in reduced concrete strength. Various researchers studied the workability of concrete with the substitution of waste glass in concrete as shown in Figure 4.

The workability of concrete increased with the substitution of waste glass as binding or as fine aggregates [65]. This is because waste glass acts as a micro filler that fills the voids between concrete ingredients, resulting in more cement paste being available for lubrication and more workable concrete. It has also been reported that the workability of concrete increases with the substitution of waste glass due to less or no water absorption [2,66]. However, some studies observed a decrease in the workability of concrete with the substitution of waste glass due to its physical nature (larger surface and rough surface texture). According to one research study, the workability of concrete reduced with waste glass owing to the higher surface area and rough surface roughness, which increased the internal friction between concrete ingredients, resulting in less workable concrete [49].

### 4.2. Density

**Figure 5:**
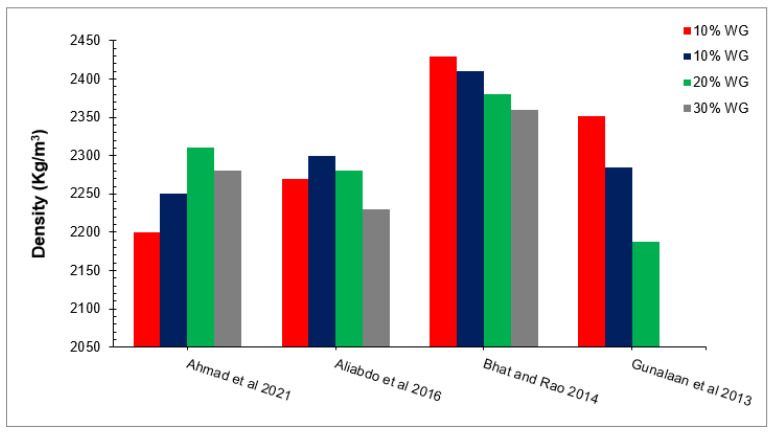
The density of concrete with different percentages of waste glass [49,60,61,67].

Concrete compressive strength primary effects on the flowability of concrete. Low flowability increased the compaction, resulting in increased pores in concrete and leading to less dense concrete, adversely affecting the compressive strength. That is why density is one of the most critical factors that must be considered during concrete mix designs. Concrete density with glass is shown in Figure 5 as per past studies [49,60,61,67]. A study reported that concrete density is 1.25% more than the reference concrete at 10% substitution of waste glass by the weight of binders [68]. However, a study reported that the density of concrete decreased with the substitution of waste glass due to the low-specific gravity value of waste glass (2.58) as compared to cement (3.15) [69]. A study also reported that density decreased with the substitution of waste glass [55]. Waste glass has also been reported to have considerably improved concrete density [60]. A study observed that the density of concrete increased as the substitution of waste glass increased up to 20% by the weight of binders and then decreased gradually [2]. The favorable influence of waste glass on density is attributed to micro filling, which fills spaces between concrete materials, resulting in denser concrete. However, a loss in workability was observed at a greater dose of waste glass (30%) due to lower flowability, which enhanced compaction affordances, resulting in more voids in the pores and a lower density of concrete [49].

## 5. Mechanical Properties

### 5.1. Compressive Strength

**Figure 6:**
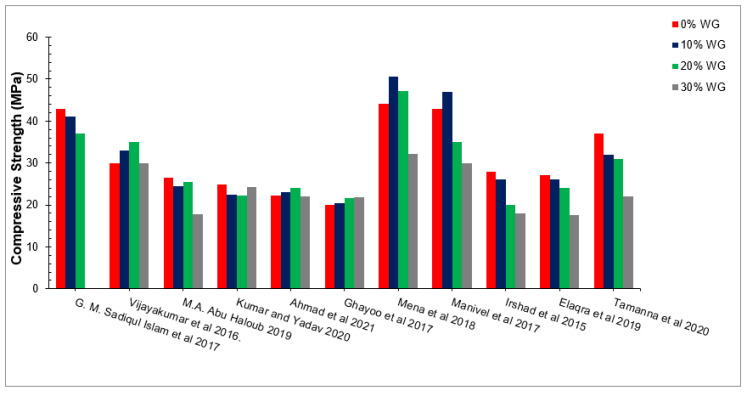
Compressive strength of concrete with different percentages of waste glass [49,56,70,71,72,73,74,75,76].

Compressive strength is one of the most significant characteristics of concrete, which provides the idea about the quality of concrete. This particular test determines whether concrete work has been performed properly or not. Figure 6 and Table 2 show the compressive strength of concrete with a partial replacement of WG as the binding material as per past studies.

A study showed that, at 10% replacements, the compressive strength increased by about 31%, 16%, and 9% at 7, 14, and 28 days of curing for the reference concrete, respectively [77]. Similarly, a study reported that compressive strengths at 7- and 28-days curing are about 3 to 4% higher than conventional concrete at 15% replacement of waste glass with cement [78]. Research was carried out on waste glass as a partial substitution of cement. The substitution ratio varies from 0% to 25% in increments of 5%. It can be observed that compressive improved considerably up to 10% substitution of waste glass [79]. A study concludes that 30% cement replacement with waste glass shows the optimum percentage of waste glass at 7 days. However, a study observed a decrease in compressive strength with glass substitution at 7 days, but a considerable improvement was observed for glass substitution after 7 days in a past study [64]. This was due to the pozzolanic reactions of waste glass, as pozzolanic reactions proceed slowly. Similar results showed that waste glass does not significantly improve in the early days (7 days). However, strength considerably improved later (28 and 56 days) [49]. It has also been reported that at 30% replacement of the waste glass, fine aggregates are 18.6% higher than for conventional concrete compressive strengths. Therefore, 40% replacement is the optimum replacement [80]. The good impact of waste glass is due to the micro filling that fills the spaces of concrete materials, resulting in denser concrete with greater strength. Furthermore, the pozzolanic reaction of waste glass produces a secondary cementitious component (CSH gel), which increases the binding characteristics of cement paste, resulting in denser concrete. The combination of micro-infill and the pozzolanic reaction of waste glass improves the compressive strength of concrete. Nevertheless, a reduction in compressive capacity was detected at a large replacement ratio of WG (more than 30% substitution of waste glass by weight of binder) due to the lower flowability, causing increased pores in concrete, which results in less compressive strength. Moreover, a study reported that, at a higher dose (more than 30% substitution of WG by weight of binder), the compressive strength of waste decreased due to the dilution impact, which produces alkali-silica reactions (ASR) [49]. 

#### Axial Stress–Strain Curve

**Figure 7:**
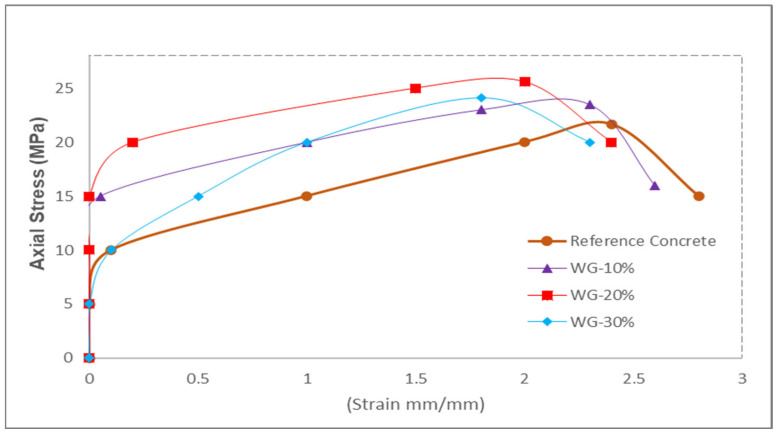
Axial stress–strain curve [56].

The stress–strain curve of different replacement ratios of WG is shown in Figure 7. The tension necessary to produce the initial strain in WG was significantly greater than the tension required in the control concrete. The secondary cementitious compound (CSH) was formed due to the pozzolanic reaction. CSH improved the binding characteristics of the paste; thus, a larger load was needed to begin strain in the paste. A further factor that contributed to the increase in the initial strain was the micro-filling effects of WG, which resulted in a more compact mass. Similarly, due to the combination of pozzolanic reactions and micro filling of WG, the ultimate stress was enhanced with the replacement of WG up to 20% and then reduced due to lower flowability. Despite the fact that the waste glass increased ultimate stresses significantly, the ultimate strain was reduced with the replacement of the WG, which resulted in the brittle failure of concrete. Hence, it is recommended that certain tensile reinforcement (fibers) could be added to increase the concrete’s tensile strength (ductile failure). As shown in Figure 7, both reference concrete and concrete made with waste exhibited approximately the same stress–strain curve in the descending portion of the curve.

**Figure 8:**
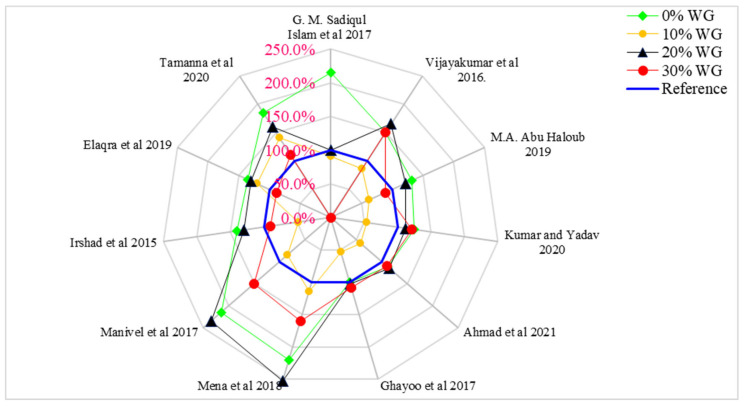
Relative compressive strength: Data source [49,56,70,71,72,73,74,75,76].

Figure 8 shows the relative compressive capacity of concrete with distinct doses of WG (0%, 10%, 20%, and 30%), in which 20 MPa compressive strength is considered the reference strength. According to Islam et al. [70], the compressive strength of concrete is approximately equal to the reference strength (20 MPa) at 20% of waste glass. Vijayakumar et al. [71] observed 65% more compressive strength than the reference strength at 20% replacement of WG. Haloub et al. [72], Kumar et al. [73], Ahmad et al. [49], and Ghayoo et al. [74] observed approximately 15% more compressive strength than the reference strength at 20% replacement of WG. The maximum compressive capacity was noted by Mena and Manivel [75], which was about 150% more than compared to the control strength. Irshad and Elaqra [72] noted 30% more compressive strength than the reference strength, while Tamanna [76] observed 85% more compressive strength at 20% substitution of waste glass compared to the reference strength. The various differences in compressive strength with respect to different researchers is due to changes in mix design, water-cement ratio, types of aggregate, temperature, and humidity, etc.

### 5.2. Split Tensile Strength

**Figure 9:**
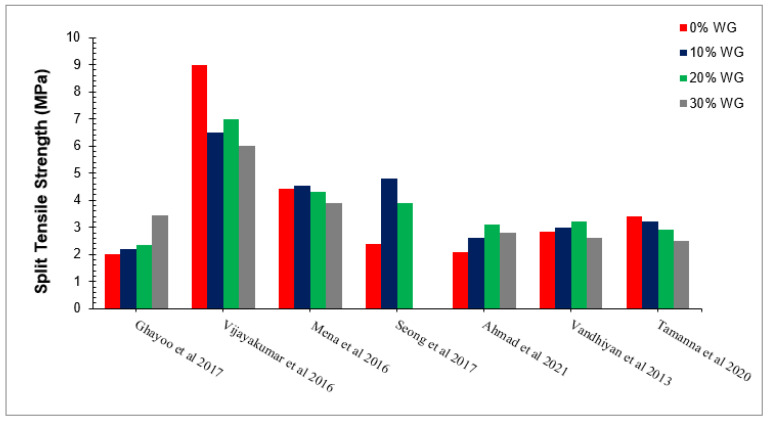
Split tensile strength of concrete with different percentages of waste glass [49,55,71,74,76,77,83].

Split tensile strength is an important test of concrete to find the tensile capacity of concrete. Lower split tensile strength of concrete will cause tensile cracks to decrease shear capacity and concrete failure in tension, resulting in brittle failure without any warning (deformation). The split tensile strength is about 10 to 15% of compressive strength. The split tensile strength of concrete with partial substitution of waste glass as per past studies is presented in Figure 9 and Table 2. A study observed a maximum split tensile strength at 20% substitution of waste glass [69]. Similar results were also reported by the researcher [81]. The research was carried out on partial cement replacement with the waste glass in concrete at 0%, 5%, 10%, and 15%. Results indicated that concrete split tensile strength was improved up to 10% replacement cement with waste glass [77]. A researcher concluded that the split tensile strength of concrete increased with up 10% substitution of waste glass and then decreased gradually, having maximum strength at 10% substitution of waste glass [60]. A study was carried out on waste glass as a partial replacement of cement at proportions of 0, 10, 20, 30, and 40% by weight of cement for M20 concrete with a water-to-binder ratio (w/c) of 0.53. It has been concluded that, at 30% replacement, the compressive split tensile strength was 4.4% higher than conventional concrete [71]. A researcher studied the effect of the partial replacement of cement with waste glass in proportion from 0 to 40% by volume at 5% interval for M20 concrete with a w/c of 0.45. It can be observed that the split tensile strength is about 19% higher than conventional concrete at 20% substitution of waste glass [69]. A researcher concluded that the tensile capacity of concrete increased 39% at 28 days of curing [78]. A study used WG as a partial substitution with cement in the percentage of 0 to 30% by the weight of cement. The results show that the tensile capacity of concrete increased up to 20% replacement of WG and decreased due to lower flowability, possessing a maximum split tensile strength at 20% substitution of waste. They can also claim that waste glass enhanced split tensile strengths more than compressive strength due to increased cement paste strength [49]. It has also been noted that concrete has less split tensile strength due to lesser cement paste strength [82]. The replacement of waste glass from secondary C-S-H, which enhanced the cement paste strength, improved the split tensile strength of concrete [49].

**Figure 10:**
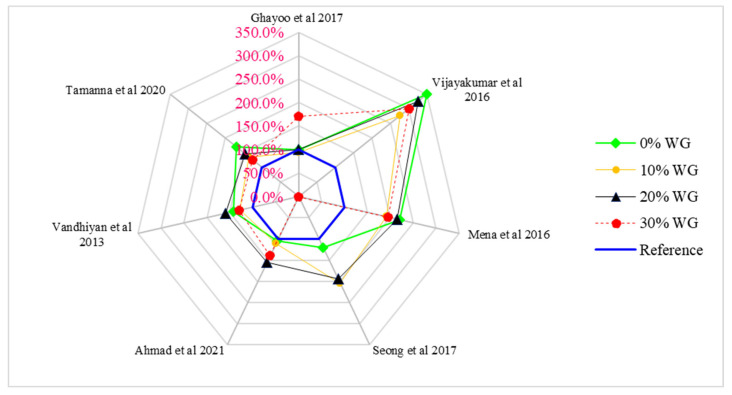
Relative analysis of split tensile strength.

Figure 10 show the relative split tensile strength of concrete with different doses of waste glass (0%, 10%, 20%, and 30%) in which 2.0 MPa split tensile strength is considered as a reference strength.

According to Ghayoo et al. [74], the split tensile strength of concrete is approximately equal to the reference strength (2.0 MPa) at 20% of waste glass. Vijayakumar et al. [71] observed 225% more split tensile strength than reference strengths at 20% substitution of waste glass. Mena et al. [75] observed approximately 115% more split tensile strength than the reference strength at 20% substitution of waste glass. Seong et al. [83] observed 95% more than compared to the reference strength. Ahmad et al. [49] Vandhiyan et al. [77] and Tamanna et al. [76] observed 55%, 60%, and 45% more split tensile strength compared to the reference strength. The various differences in compressive strength with respect to different researchers are due to changes in mix design, water-cement ratio, and environmental aspects such as temperature and humidity, etc. It can also be observed that waste glass improved compressive strengths more effectively than split tensile strength. Therefore, we recommend adding some tensile reinforcements to enhance the tensile capacities of concrete before its practical application.

## 6. Durability

### 6.1. Water Absorption

**Figure 11:**
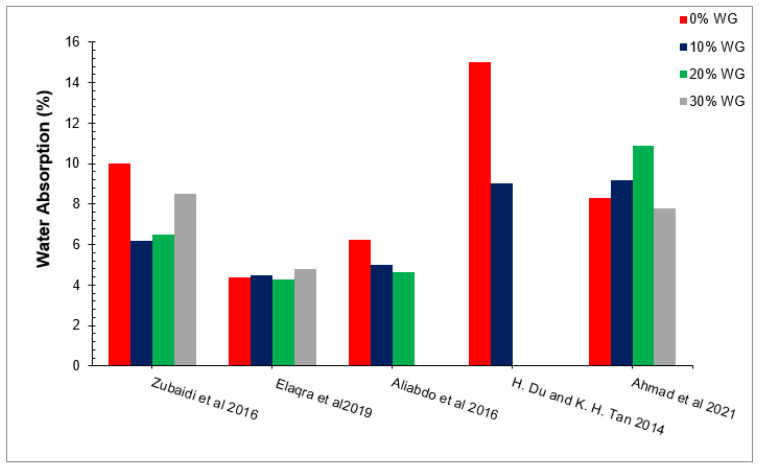
Water absorption [49,60,72,89,90].

Water absorption tests can be used to determine the durability of concrete. Water includes a toxic chemical that, when combined with other chemicals contained in cement concrete, can cause concrete disintegration. Furthermore, water absorption generates freeze and thaw actions, which add stress to the surrounding concrete, resulting in less durable concrete. According to one study, water absorption is one of the reasons for alkali–silica reactions [88]. Figure 11 shows the water absorption of concrete with varying doses of waste glass as per past studies [49,60,72,89,90]. It can be observed that waste glass decreased the water absorption of concrete due to micro filling and pozzolanic reactions [49]. A study concluded that water absorption decreased as the percentages of waste glass increased, having minimum water absorption at 10% substitution of waste glass [80]. A study observed that the water absorption of concrete decreased compared to the reference concrete for both grades of concrete (m20 and m30) [79]. However, a study observed that an increase in porosity was observed as the percentages of waste glass increased. The increase in porosity results in greater water absorption [91]. A study concluded that waste glass as cement replacement decreased porosity, resulting in more water absorption [92]. According to one study, the highest water absorption of concrete was recorded at 0% substitution of waste glass. The smallest water absorption of concrete was observed at 20% substitution of waste glass. The decrease in water absorption of concrete with waste glass replacement is due to the pozzolanic reaction of waste glass, which improves the binding qualities of cement paste. Furthermore, the micro filling of waste glass results in more dense concrete filling the voids between concrete ingredients, resulting in less water absorption. However, an increase in water absorption was observed at a greater dose of waste glass (30%) due to a lack of workability, which increased compaction efforts, resulting in more voids in hardened concrete and in more water absorption [49]. As a result, a higher dose of superplasticizer is required for a higher dose of waste glass.

### 6.2. Dry Shrinkage

Drying shrinkage is referred to as the contraction of a hardened concrete mixture as a result of a loss of capillary water. As concrete shrinks, it experiences an increase in tensile stress, which may cause cracking, internal warping and external deflection before any loading occurs. Concrete made with Portland cement undergoes drying shrinkage or a change in hydraulic volume with time. In the design of a structure, an engineer should pay attention to hydraulic volume changes in concrete. Drying shrinkage can occur in a range of areas including slabs, beams, columns, bearing walls, prestressed members, tanks, and foundations.

Several factors contribute to drying shrinkage. These factors include properties of the components, proportions of the components, mixing method, amount of moisture during curing, dry environment, and size of the member. The main reason for drying shrinkage is due to the reduction in capillary water by evaporation and the presence of water in cement paste during the drying process. The greater the amount of water present in fresh concrete, the greater the shrinkage phenomenon during drying. Several aspects influence the shrinkage potential of concrete, including the amount of mixing, the elapsed time after the addition of water, temperature fluctuations, slumping, placement, and curing. It is also important to consider the composition of the concrete. The shrinkage of concrete varies depending on the type of aggregate and cement used, and each contributes to shrinkage differently from the others. During mixing, the amount of water and additives used in concrete has a direct and indirect effect on the drying shrinkage of concrete. It is believed that concrete shrinkage is largely caused by the evaporation of the capillary water that mixes the concrete. 

**Figure 12:**
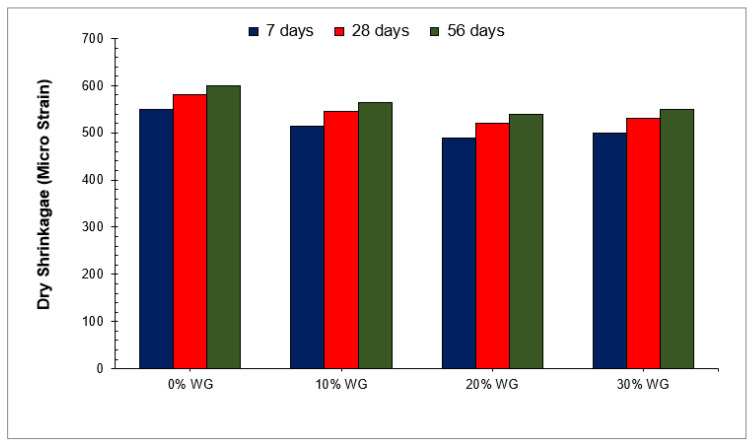
Dry shrinkage [49].

A dry shrinkage test may also be used to determine the durability of concrete. Water and other potentially dangerous compounds can quickly enter the concrete through microscopic surface cracks created by dry shrinkage. The availability of internal water, aggregate types, and voids in concrete all influence dry shrinkage. Generally, the dry shrinkage of waste glass is less than conventional concrete. A study reported that concrete with 20% waste glass as a cement replacement shows 50% less dry shrinkage [93]. Similarly, Figure 12 shows the minimum dry shrinkage at 20% substitution of waste glass [49]. It is due to the stiff nature of waste glass and the rough surface texture, which is strongly interlocked with paste. However, due to the larger particle size (more than 200 mm), internal voids increased the dry shrinkage, particularly at high temperatures. Therefore, it is recommended that finer used waste glass should be used, possessing a particle size of fewer than 75 microns. A study also reported that finer waste glass improved the performance of concrete more effectively than larger particles due to micro filling and pozzolanic reaction [49]. Research also discovered that waste glass reduces drying shrinkage by filling holes in concrete materials, which improves the internal compactness of concrete [55]. It was also shown that dry shrinkage after 28 days (56 and 90 days) was almost equivalent to dry shrinkage at 28 days. It has also been observed that the dry shrinkage rate is higher in the first 7 days and decreases or remains constant with age [88]. The previous study has shown that coarse aggregate inhibits dry shrinkage, and dry shrinkage is caused mostly by the movement of cement mortar [55]. The production of secondary C-S-H due to the substitution of waste glass increased the viscosity of cement paste, which reduced the mobility of the paste. Furthermore, the use of waste glass reduced the heat of hydration, which reduced the evaporation rate of water from the concrete’s surface, resulting in lower dry shrinkage cracks [49]. A study also shows that dry shrinkage is due to the evaporation of internal water rather than surface water [2]. The density of concrete depends upon the consistency of the material; materials such as geopolymer when used in concrete do not have well-enclosed pores, and due to these pores, water losses occur and in less than 90 days, major shrinkage occurs. For that reason, highly fine base materials result in shrinkages stresses and high strain.

Nevertheless, the WG powder, due to its micro filler property, decreases the size of the pore, and the internal water in concrete remains internally. The shrinkage due to the loss of water minimizes and the shrinkage in volume decreases [94]. Moreover, the surface bond strength between WG particles is much higher than in the cement particle [95]. Therefore, the shrinkage in the volume of concrete in a hardened state is much lesser than a volumetric shrinkage in concrete without WG powder. The rising curing temperature reduces the drying shrinkage and provides a much closer and well-compacted particle structure geopolymer [2]. The drying shrinkage should be minimized to the ultimate level for better mechanical properties and high weather resistance. As the non-reactive material is not subject to shrinkage, an excessive amount of WG can be used in geopolymer concrete and provide much higher resistance and stability against shrinkage [95]. Although there is a difference in temperature found between the laboratory and outside in open environments, which can mislead the result and performance of the WG percentage of usage and many other factors. It is recommended that a study should be conducted in which practice environmental conditions and factors should be applied to obtain information on long-term performance [96].

## 7. Advantages of Waste Glass in Concrete

The environment is protected from dumping and the landfills of WG [57]. PC and WG concretes were studied for their environmental impact by Hilton et al. [97]. The result showed that more than 13% of environmental impact was reduced with a comparison is carried out between WG and PC concrete. Moreover, by the use of WG concrete, the emission of harmful gases produced by PC concrete decreased by 20%, which contributed to decreasing global warming from 0.17 to 0.42 g CO_2_/g, and it is produced by glass-based cement, which reduces 83% of CO_2_ emission when using concrete consisting of OPC [98]. A similar outcome was achieved by Patel et al. [99] in their research. The application of granulated foam glass (GFG) in concrete might significantly reduce the volume of waste glass and enhanced the recycling industry in improving environmental performance [100]. WHO reported that many environmental issues such as acid rain, ozone depletion, photochemical instability, and WGP are reduced by using WG in concrete instead of using OPC in concrete production. There are many other benefits associated with the use of WG in concrete. The first is reducing landfill problems when waste is reused. Secondly, WG is used in concrete as an admixture and will be added to concrete, and concrete will lose some natural material. Therefore, the natural material is also saved by the use of WG. However, WG base cement is still not investigated completely as there are still some unanswered questions to the property of WG cement, such as long-term serviceability, impact assessment, and carbon footprints.

Glass is a material that takes the maximum time for biodegradability when used in landfills. This property of glass makes it a huge problem in the waste management sector throughout the world [49]. Moreover, cement factories are facing continuous discrimination and resistance from environmental protection agencies by using natural resources in the manifestations of cement. Using natural resources gives rise to many environmental problems such as climate change, the greenhouse effect, and many more. The usage of glass waste in the construction industry will provide solutions to many environmental problems and also will boost the country’s economy. The size particle of waste glass provided a significant result in a review study mentioning that glass plays an important role in ASR destruction in concrete performance. The pozzolanic property of waste glass renders its use in concrete more beneficial with a particle size of 100 μm. Many types of research studies show that increasing the percentage of glass waste in concrete leads to a reduction in concrete performance. Therefore, detailed study and research are needed to address the optimum value of glass powder usage in concrete as a replacement. The correct size to control the shrinkage property of concrete is needed. As cement is a mixture of raw materials under different processes and glass possesses pozzolanic behaviors, the study on the use of waste glass in cement manufacturing is also required.

## 8. Disadvantages of Waste Glass in Concrete (Alkali-Silica Reaction)

One of the major disadvantages of using waste glass in concrete is the alkali–silica reaction (ASR). The availability of significant silica in waste glass is a key difficulty because alkali-concentrated cement produces ASR, leading to expansive gel formations [101]. A researcher studies the effect of particle size of waste glass as coarse aggregate on the expansion due to the ASR of concrete. The particle size of waste was 4.75 to 0.15 mm in different percentage ratios starting from 0 up to 100%. The result concludes that the expansion due to ASR increased as the particle size of waste glass increased. A minimum expansion was observed at 20% substitution of waste glass with a particle size of 0.15 mm. The optimum percentage (20%) and particle size less than 1.180 mm does not show any harmful effects of ASR [102]. It has also been reported that waste glass with particle sizes less than 1 mm does show expansion due to ASR at 20% substitution of waste glass. Furthermore, it also suggests that waste glass with particle size less than 150 µm can be used up to 40% without any negative effects on the performance of concrete [96]. A researcher observed that waste glass possessing particles less than 50 mm can be safely used up to 70% without any negative effects on the performance of concrete [103]. A study concludes that the pozzolanic activity of waste glass starts from particle sizes less than 300 mm and increases by decreasing the particle size of waste glass. The maximum pozzolanic value was observed at a particle size of 45 mm. Moreover, waste glass can use up to 20% replacement of cement without any effect on the mechanical performance of concrete [49]. Furthermore, there was no expansion due to ASR up to 20% substitution of waste glass.

**Figure 13:**
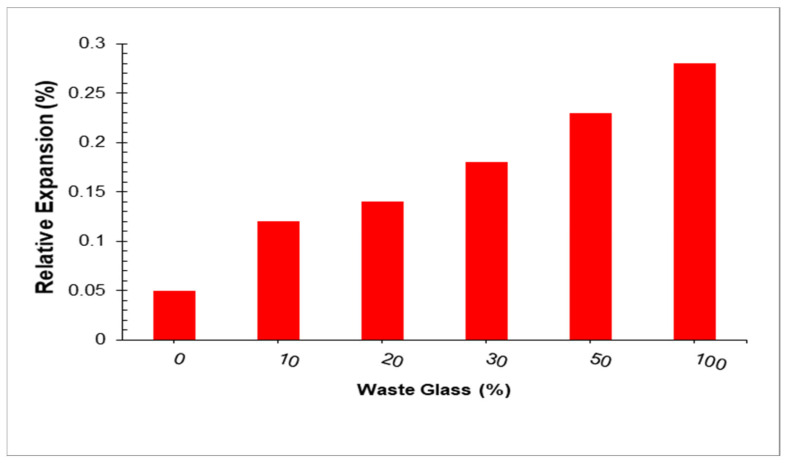
Relative expansion with waste glass [110].

The ASR gel creates expanding strains along the reaction zone that may exceed the concrete’s tensile strength restriction, allowing cracks to arise. As a result, an extra opportunity for penetration and absorption of the external solution is produced, lowering the durability. Serpa et al. [104] used Portuguese recycled glass to partially substitute natural fine aggregate in mortars at values of 0%, 5%, and 20% by weight. The findings indicate that substituting natural fine aggregate with waste glass did not result in higher ASR expansion at 14 days but did result in a modest rise at 28 days. As glass content increased, expansion also increased. Limbachiya et al. [105] investigated the ASR expansion of concretes incorporating mixed color beverage glass (size 5 mm) as a natural sand replacement at levels of 0%, 5%, 10%, 15%, and 20% by weight at ages 3, 7, and 14 days. According to the findings, ASR expansion increased as the glass sand content increased [104]. Idir et al. [106] investigated the ASR expansion of mortars by incorporating waste glass as a natural fine aggregate replacement at 20% and 40% weight ratios. Kou and Poon [107] used cullet waste beverage glass bottles (size 5 mm) to partially substitute sand in mortars at weight levels of 0, 15, 30, and 45%. ASR expansion increased with increasing glass sand content. Oliveira et al. [108] reported that ASR expansion of concretes containing amber glass (maximum particle size 4.76 mm) as natural sand replacements increased with increasing glass sand content. Ling and Poon [109] reported an increase in the ASR expansion of mortar containing 100% beverage waste glass (size < 5 mm) as fine aggregates. According to Park et al. [110], the expansion rate measured via ASR expansion in line with ASTM C 1260 [111] increased with increasing emerald, green waste glass (size 5 mm). Figure 13 shows that the relative expansion with waste glass is reported by Park et al. [110]. With the addition of 10, 20, 30, 50, and 100% glass sand, the expansion of mortar specimens increased by approximately 85.7%, 116.67%, 159.76%, 228.5%, and 285.71%, respectively. Ling et al. [112] found that using waste glass (about 23% coarser particles 10–5 mm) as a natural sand substitute at 25%, 50%, 75%, and 100% by weight increased the ASR expansion of mortars. With increasing glass sand content, ASR growth increased. Shayan and Xu [113] used recycled waste glass (size 4.75–0.15 mm) to replace natural fine aggregate in mortars at amounts ranging from 0% to 100% by weight, with a 10% increment. The ASR expansion data revealed that the higher the glass concentration in mortar bars, the greater the expansion. Jin et al. [114] investigated the ASR expansion of mortars by incorporating transparent soda-lime glass as a natural sand replacement at amounts ranging from 0% to 100% with a 10% increment by weight for up to 14 days. With increased glass sand contents, ASR expanded further. The ASR expansion of mortars incorporating waste glass as a natural fine aggregate replacement was investigated by Topcu et al. [115]. Waste glass (size 4.75–0.3 mm) was substituted by natural sand. With increased glass sand contents, ASR expanded further at the ages of 14 and 21 days, Degirmenci et al. [116] investigated the ASR growth of mortars containing 10, 30, and 100% glass sand. The growth of ASR increased as glass sand content increased. Ismail and Al-Hashmi [65] used crushed waste glass (size 4.75–0.15 mm) to partially substitute natural sand in concrete at weight levels of 0, 10, 15, and 20%. According to the findings, the presence of discarded glass lowered ASR growth. As the volume of discarded glass increased, ASR decrease. Glass sand content has a substantial influence on ASR growth.

As seen from the preceding discussion in this section, ASR growth increased with increasing glass sand content. The increase in expansion is due to the 100% glass sand having many amorphous ASR products, as shown in Figure 13, although the gels were mostly found at the interfaces between the glass particles [104]. As Dhir et al. [51] pointed out, the dissolution and superficial leaching of the glass silica and the creation of a silica gel surface around the aggregate accounted for the expansion evolution over time. According to Corinaldesi et al. [117], no ASR expansion was identified with glass particles with particle sizes up to 100 microns meter, even when largely substituted with natural sand at levels of 30 and 70% by weight, indicating the viability of waste glass reuse as fine aggregates in mortar and concrete. The ASR expansion of mortars containing varying fineness of mixed color waste glass was investigated by Idir et al. [96]. By weight, 20% and 40% of the marble sand was substituted with discarded glass. The utilized waste glass had a diameter of up to 7.8 microns meter. With increasing waste glass fineness, ASR expansion was reduced. Idir et al. [118] found that with increasing glass sand specific surface area, the ASR expansion of mortars containing mixed color glass (40% colorless, 33% yellow, 20% green, 15 blue, and 6% impurities) at levels of 20 and 40% decreased. No swelling due to ASR was detected if glass grains were smaller than 1 mm. Fine glass powders with specific surface areas ranging from 180 m^2^/kg to 540 m^2^/kg were also shown to minimize the expansions of mortars subjected to ASR, according to the researchers [49]. Du and Tan [119] used several colors of waste glass to partially substitute natural sand in mortars at quantities ranging from 0 to 25%. Particle sizes ranged from 2.36 to 1.18 mm, 0.6 to 0.3 mm, and 0.15 mm for each color. With increasing glass size, independent of glass color, ASR growth increased. The highest and smallest ASR expansions were observed at 2.36 mm and 0.15 mm glass sand sizes, respectively. Using finer WG powders instead of coarse glass aggregates, ASR gel formation can be reduced. Researchers estimate 1–1.18 mm as the essential particle size of WG powder [120]. However, other studies have shown a safe limit of 0.6 mm particle size [121]. The previous study [117] found that replacing 70% of fine aggregates with 36–50 μm WG powder particles in concrete did not cause detrimental ASR growth.

Furthermore, researchers found that glass sand with a grain size of less than 4.5 mm and no surface defects did not generate expanding ASR gels at a sand replacement level of up to 40% [76]. The presence of microcracks in the particle is undesirable because they produce pores and store solutions for subsequent reactions, increasing ASR reactivity. This guarantees that the ASR risk is not mainly influenced by particle size; other parameters impacting ASR gel formation include the concentration of WG, the kind of cement and aggregates, the mix ratio, and the water–cement ratio of the concrete mix. As a result, based on the chemical characteristics of WG and maintaining an optimal degree of replacement and particle size, it is possible to reduce the danger of ASR. Well-graded WG powders can increase densities due to micro filling while lowering ASR expansion. Furthermore, the presence of lithium ions inhibits growth by altering the composition of the ASR gel [122]. Table 3 shows the summary of the researcher’s regarding ASR associated with the use of waste glass in concrete.

## 9. Conclusions

This review highlights a reduction in glass waste problems in nature, and current research progress is discussed. The properties such as the strength (compression and tension) of concrete using glass waste are a major concern to know in this research area. The detailed conclusion is given below.

- The decreased in flowability of concrete with waste glass is mainly due to the sharper and larger surface area of waste glass. However, it can be controlled by using a superplasticizer.
- The chemical composition of waste glass is similar to clay, which indicates that it can be used as a raw material for cement production without any negative effects on cement’s physical and chemical properties. However, there is a gap in knowledge in this regard, and further research is recommended before practical applications.
- Mechanical performance and durability aspects of waste glass mainly depend on particle size and optimum dose. Fine particles of waste glass improve mechanical performance and durability, while coarse particles decrease concrete’s mechanical and durability performance. The particle of waste glassless 4.75 mm can be used without any negative effects on concrete’s mechanical and durability performance. However, for high strength, it is suggested to use glass particles that are less than 100 µm.
- The optimum dose of waste glass also plays an important on the mechanical and durability performance of concrete. The optimum dose depends on various parameters such as particle size, mix design, types of glass, chemical, and physical aspects.
- It can also be observed that the careful selection of waste glass particle size and optimum dose improved concrete’s mechanical and durability performance considerably.

The overall study demonstrates that waste can be used successfully in concrete as binding materials or as a fine aggregate. The utilization of waste glass in concrete provides multiple benefits, including problems of natural resources presentation, ecofriendly environment, low cost, air pollution problems, and waste management problems without any negative effects on the mechanical and durability performance of concrete provided that optimum doses and particles size are correctly used. However, less information is available on the durability aspects of concrete with waste glass. Furthermore, although waste glass improved the mechanical performance of concrete, it still has lower tensile capacities, leading to brittle failure. Therefore, further research was recommended to enhance the tensile capacity of concrete with the addition of fibers.

## Figures and Tables

**Table 1:** Chemical composition of waste glass [49,57,58,59].

Chemical Name	Percentages
SiO_2_	70 to 75
CaO	8 to 10
Al_2_O_3_	1 to 3
MgO	1.5 to 3%
NaO_2_	0 to 15%
Fe_2_O_3_	0.5 to 1%
SO_3_	0.3 to 0.80
K_2_O	0.25 to 1.4
ZnO	0.02 to 1.5
NiO	0.07 to 1.0
SrO	0.94 to 1.8
CI	0.61 to 0.92
P_2_O_5_	0.01 to 0.25

**Table 2:** Summary of mechanical performance of concrete with waste glass.

Authors/Reference	Waste Glass (%)	Compressive Strength (%)	Split Tensile Strength (%)
Hussian et al. [84]	15%	17 +	
Haloub et al. [72]	20%	4.0 −	
Ahmad et al. [49]	20%	7.6 +	47 +
Prudhvi et al. [85]	16%	25 +	
Islam et al. [70]	20%	Equal to control	
Vijayakumar et al. [71]	20%	16 +	
Harish et al. [86]	30%	15 +	
Ghayoo et al. [74]	20%	8.0 +	55 +
Vandhiyan et al. [87]	10%	9.0 +	
Mena and Manivel [75]	20%	7.0 +	Equal to control
Irshad and Elaqra [72]	20%	11 +	
Sakale et al.	20%	24 +	

+ Increased and − decreased.

**Table 3:** Summary of researchers regarding ASR associated with the use of waste glass in concrete.

Authors/References	Remarks
Guo et al. [123]	There is no ASR expansion when the median diameter of glass particles is finer than 600 µm in concrete.
Khan et al. [124]	Concrete with alkali-activated GGBFS concrete specimens with waste glass aggregate experienced patterns or mapping-type cracks on the surface, which was typically found in the ASR-affected concrete. However, no such cracks occurred in the alkali-activated FA or the FA-concrete specimens.
Ling et al. [109]	All ASR expansion results of the tested samples were below 0.2% on the 14th day. Decreasing the maximum particle size of the glass from 5 mm to 600 mm effectively reduced the risk of ASR.
Idir et al. [118]	Proposed that the critical threshold of glass particle size was around 0.9–1 mm.
Hamau [125]	ASR expansion could be neglected for glass with a particles size smaller than 1 mm. However, when the particle size is larger than 1.25 mm, significant expansion could occur when the mortar prisms were filled with 20% glass particles.
Sun et al. [126]	The expansion increased significantly after the chemical activation on 10% WGP. However, this phenomenon was undiscovered for the 300 μm WGP sample, which might be because the gels were difficult to accumulate on the surface of coarse waste glass due to its relatively low activity.
Rajabipour et al. [127]	Use of GP less than 75 μm in size leads to reduced ASR value.
Shutt et al. [128]	Particularly, it was reported that ground soda-lime glass with dimension < 300 µm can safely be introduced in concrete.
Belouadah et al. [129]	Most glass powder particles are smaller than 20 µm, which may contribute to activating its pozzolanic activity and reducing the risk of alkali–silica reaction (ASR).
Sadowski et al. [130]	Laboratory results show that the use of an air-entraining agent is an effective method to reduce or eliminate the ASR expansion since the expansive reaction products can permeate into additional pores.
Afshinnia et al. [131], Aliabdo et al. [60], Rodier et al. [132], Metwally et al. [133], Figueira et al. [134], Maraghechi et al. [135], Meyer et al. [136], Pereira et al. [137], Ali et al. [138], Topcu et al. [139], Dyer et al. [140], Schwarz et al. [141], Zheng et al. [142], Li et al. [143].	For particles finer than 300 µm, other processes that depend on surface-to-volume ratios become predominant and the expansion decreases with greater fineness.

## Data Availability

All Data are available in the manuscript.
